# Colloid Metals

**Published:** 1914-05-02

**Authors:** 


### MODERN METHODS.

### Colloid Metals.

Some instructive remarks from the pen of Dr. Fortescue-Brickdale are published in the March issue of the *Bristol Medico-Chirurgical Journal* on the therapeutic actions of metals in the colloid state. Some of these substances are being very extensively used just now for a variety of diseases; and it is important that the medical profession should have a clear idea of their merits and their dangers.

The colloid state now connotes a condition of matter in the form of exceedingly fine particles which can be suspended in a fluid medium and then exhibit certain definite properties. The particles are small enough to pass through an ordinary filter, but large enough to be visible by means of the ultra-microscope. Such colloid solutions stand midway between an ordinary emulsion or suspension and a true molecular solution. The particles are in a state of perpetual movement: "Brownian." in some cases but not in all. Colloid metals inhibit the growth of bacteria, though Dr. Fortescue-Brickdale confesses that he does not know why: in other words, they are strongly antiseptic, and some colloid metals are distinctly more so than others. When a sufficient dose of one of these antiseptic colloids is introduced into the body of a person suffering from a septic infection there is a severe reaction. Temperature rises, the pulse rate increases, cyanosis, leucocytosis, and other signs of intoxication develop. It is supposed that these are the result of the death of large numbers of the invading micro-organisms, and the setting free of morbid products from their corpses. In a healthy person the reaction is much less acute, in fact scarcely noticeable. The result of these observations is that various colloid metals have been used in the treatment of septic infections of diverse kinds, especially in France, whence most of the literature of this treatment so far available for study has been derived.

Colloid silver, for instance, has been injected into the pleura and the bladder in septic inflammations of those parts. It has also been given intravenously for pelvic suppuration, septicæmia, and other conditions, and has been used as a local application to the throat and nose. Under the name of electrargol it is manufactured and sold by a well-known firm of French wholesale druggists in convenient form. It has also been given by inunction for pneumonia and broncho-pneumonia in children, and occasionally also hypodermically or intravenously for these conditions. Infective endocarditis, rheumatism, and diphtheria have also been thus treated. By the mouth it has been given in typhoid fever and dysentery. One case at least of chronic silver poisoning has been published.

Colloid mercury has been used for syphilitic manifestations, both locally and by intravenous injection. Colloid iron is being used for the treatment of anæmias, primary and secondary. It is also stated that the subcutaneous injection of colloid iron is beneficial in cases of erysipelas and cellulitis. Colloid sulphur has been employed for chronic and subacute rheumatoid arthritis. Colloid copper is being used for cancer and for tuberculosis. One essential precaution is to abstain from boiling the solutions before injecting them.

It will be seen that diseases of many different kinds are being treated by colloid metals just now, and that most of them are of a particularly rebellious character. Of the results it is perhaps too early to speak very definitely. Certainly some encouraging reports are to hand of septicæmia, but then the same is true of any new treatment for this serious disease, in which recovery is not always due to the particular line of treatment that has been followed, though it is naturally ascribed to the latter. Colloid copper has been given a fair and thorough trial for cancer at one well-known institution, and has been abandoned as not merely disappointing but positively dangerous. "The most diabolical treatment I have ever seen," was the verdict of an experienced ward sister on these injections, and the medical officers were equally decided about the results. Symptoms were made much worse, and the downward progress of the cases was greatly accelerated. Some optimistic reports have been published, but there seems very little real foundation for them.

Enthusiasm always greets a new remedy concerning which large claims are made, and something has to be allowed for this attitude of mind in analysing the first reports of the therapeutics of colloids. As Dr. Fortescue-Brickdale very truly and pointedly remarks, successes are more often published than failures; and even when the latter are reported in the medical press the manufacturers are not assiduous in circulating them among the medical profession. It is safe to say that a very liberal discount must be allowed off the optimistic reports from France and elsewhere, and that it is still in doubt whether colloids have any real therapeutic value at all. Certainly if they have it is in septic infections, and not in such diseases as cancer and tuberculosis. The colloid metals are not by any means free from danger. Their indiscriminate use without the greatest care being exercised will almost certainly lead to disasters; it has already led to some, though the majority have gone unrecorded. Clinicians will do well to bear these facts in mind before rushing to take up colloid treatment on the slightest provocation. There are indications that an attempt is being made to constitute this the latest "fad" of the medical moment, and a word of warning may therefore be of use.

